# Characterization and Assessment of PEEK/Silicon Dioxide Composite

**DOI:** 10.1155/2023/3343071

**Published:** 2023-01-03

**Authors:** Husam Mohammed Saeed, Raghdaa Kareem Jassim

**Affiliations:** Department of Prosthodontics, College of Dentistry, University of Baghdad, Baghdad, Iraq

## Abstract

**Background:**

In the domain of dentistry, PEEK materials have been utilised as removable partial denture frameworks, implant substances, and fixed dental prostheses. Even though PEEK polymer has a low modulus of elasticity, its mechanical attributes could be fine-tuned by integrating various inorganic filler materials. The study intends to characterise and assess Nano SiO_2_/PEEK composite prepared by using the melt blend approach.

**Materials and Methods:**

The design of this study uses 3% SiO_2_ and PEEK composite made using the melt blend technique and studies their characteristics with reference to pure PEEK. The following assessments are conducted: SEM and EDX assessment with AFM investigation in addition to tensile strength test, transverse strength test, and wettability test. All data were scrutinised by SPSS software version 24 and the statistical analyses included the mean, standard deviation, and the independent sample *t*-test.

**Results:**

The outcomes of this investigation pertain to the differences in characteristics of a composite of SiO_2_/PEEK compared with pure PEEK. The outcomes indicate that there is a statistically highly significant increase in the mean value of transverse strength was seen with the PEEK/SiO_2_ composite (3503.02 MPa) versus PEEK (2694.61 MPa), while there is a statistically significant decrease in the mean value of the tensile strength for PEEK/SiO_2_ (63.69 MPa) versus PEEK (97.62 MPa). Moreover, improvement in hydrophobic characteristics and surface roughness of PEEK/SiO_2_ (81.78°), (0.66 nm) versus PEEK (71.01°), (1.23 nm), respectively, thus giving more chance to composite to be investigated in human bone/implant substitution. Furthermore, the results of EDX and SEM images exhibited adequate distribution of Nano SiO_2_ within the PEEK matrix. There was also a statistically substantial decrease in the surface roughness and tensile strength obtained from the AFM investigation.

**Conclusion:**

As far as this study is concerned, a conclusion can be made that we can use 3% Nano SiO_2_ to prepare a composite of SiO_2_/PEEK by using the melt blend approach. Nano SiO_2_ can alter the SiO_2_/PEEK composite's transverse strength and reduce the hydrophobic characteristics of the surfaces with proper distribution of nanoparticles within the matrix of PEEK with less surface roughness.

## 1. Introduction

The PEEK (poly ether-ether-ketone) is derived from poly aryl-ether-ketones. PEEK is identified chemically as a linear configuration of poly aryl-ether-ketone. PEEK is a high-performance, semicrystalline thermoplastic polymeric substance and it is a melt processable aromatic polymer; the Tm (melting point) lies between 330°C and 385°C, which depends on the relative number of ether-ketone groups connecting the phenylene rings. The extent of crystallinity is dependent on the thermal histories as well as the processing conditions, like annealing treatments and cooling rates. PEEK has attracted interest as a medical transplant material since it has excellent biocompatibility, dimensional stability, resistance to chemicals, and mechanical attributes and displays a low modulus like that of human bone [1].

In the dentistry domain, PEEK materials have been examined for use as fixed dental prostheses or detachable partial denture frameworks. Even though PEEK is a polymer with low modulus, its mechanical qualities can be regulated by including inorganic fillers and modifying the filler content. Furthermore, bioactive materials have been recently introduced to combine the strength of composites and the benefits of their bioactivity. Moreover, for commercial uses, the primary aim of including inorganic fillers into polymers is to reduce cost and improve stiffness [2–4].

Cortical bone, titanium (Ti), and ceramics have greater elastic modulus compared to PEEK material. PEEK exhibits elastic modulus (3–4 GPa), high stability, and low density (1.32 g/cm^3^). In the dentistry field, the implants must have greater elastic modulus, which is provided by PEEK material, specifically for abutments and superstructures [5].

A number of strengthened composites have been formed using PEEK: CFR (carbon-fibre reinforced) PEEK and GFR (glass-fibre reinforced) PEEK. The value of the elastic modulus of both these materials can reach up to 18 GPa. Osseointegration targeted studies compare PEEK and conventional implant candidates, such as titanium and zirconium. Research studies report insignificant differences [6, 7].

PEEK is classified as a poly-aryl-ether-ketone (PAEK) polymer. It is used as a substitute of metal implants for orthopaedic and trauma-related uses. It is an aromatic compound containing ketone (-CO-) and ether (-O-) groups in the centre of the aryl rings. It is a white radiolucent material that stays thermally stable up to temperatures of 335.8°C. It does not affect oral mucosa and demonstrates a low plaque affinity. Its attributes include heat conductivity of 0.29 W/mK, 1140–170 MPa flexural modulus, and 300 kg/m^3^ density [7].

Cook et al. fortified the PEEK material implant with titanium and carbon fibre and then placed it into femurs. After the evaluation of 8 weeks, similar contact ratios for bone-implant were reported. [8].

Nanocomposites of this polymer have attracted substantial interest and attention globally during the previous decade. Among the various fabrication techniques for polymer Nanocomposites building, the sol-gel technique seems to be the most beneficial one.

Initially, the Nanoparticles were dispersed and further combined at molecular or near-molecular level with the polymer gel [9].

Two primary approaches have been used to address the issue of PEEK's inert nature. One method is a surface adjustment to activate PEEK using only the surface treatment or treatment combined with a coating on the surface. Another approach is to prepare bioactive composites of PEEK by introducing bioactive substances into PEEK material. Researchers believe that altered bioactive PEEK material will have a broad range of orthopaedic uses [10].

The addition of SiO_2_ particles improves the heterogeneous crystallisation of the PEEK matrix. The substantial improvement in thermal and mechanical properties of the PEEK composites indicates that these composites can prove to be useful for uses in electronic packaging using Nanosubstrates. Most PEEK materials used in dentistry are reinforced by inorganic filler material to enhance their rigidity [11, 12].

In 2019, Rikitoku et al. carried out research to evaluate how the different levels of SiO_2_ in the PEEK material affected the bonding between resin cement and PEEK. The study assessed TBS (tensile bond strength) prior to and after 10,000 thermal cycles besides flexural strength, flexural modulus, and crystallinity. Furthermore, the tensile bond strength was increased and the greatest flexural strength was obtained when SiO_2_ content was increased in PEEK material at 40 wt. % SiO_2_ [13].

Further research using composites of PEEK material reinforced by Nanosized Al_2_O_3_ and SiO_2_ particulates indicate an enhancement in the rigidity, tensile strength, and transverse strength. The addition of the inorganic filler material into the PEEK matrix can enhance the thermal stability of the resultant Nanocomposites [9].

The aim of this study is to modify and improve the physical and mechanical properties of PEEK by an additional 3% wt. Nano SiO_2_. The null hypothesis entails that the addition of Nano SiO_2_ will not affect PEEK properties.

## 2. Materials and Methods

**Figure 1:**
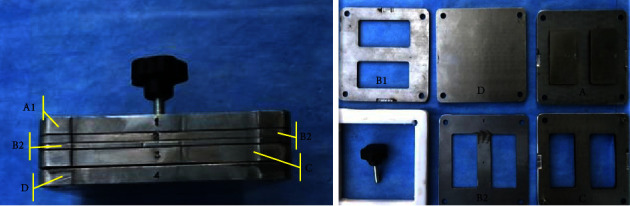
Parts of mould.

This research used Nanosilicon dioxide powder (SiO_2_) in pulverised form, coated with 3–4% KH570 Silane coupling agent. Nanomaterials particle size 20–30 nm Silane coupling agent KH570 is gamma Methacryloxy propyl trimethoxy silane (CAS No: 2530-85-0). Houston Inc., USA. This Nano will be added to PEEK powder (China type KLC607) of microsized with an average size of 68.35 *μ*m. Based on the research strategy, a mould was formed, as displayed in Figure 1. Compression moulding was performed using this mould to create samples required for the assessment.

### 2.1. Preparation of SiO_2_/PEEK Composite Mixture

At the beginning of this stage, Nano SiO_2_ (3%, 2.1 gm) is added to alcohol and dissolved using a probe sonicator (Ultrasonic-Bandilin-Sonoplus, Germany) with 80 pw and frequency of 000.5 s for 15 minutes with constant cooling. Then, powdered PEEK (97% wt. 67.9 gm) is gradually added to alcohol which contains SiO_2_. This concoction is then blended by a rotatory mechanical stirrer (Urostar power-Bika-werk, Germany) for 15 minutes at room temperature [14]. Furthermore, the SiO_2_/PEEK blend was dehydrated in a hot air oven for 2 hours at 150°C as per Kurtz and Devine, 2007 [15].

A thermal compression and melt blend technique was an appropriate method using which Nano SiO_2_/PEEK composites were prepared. Usually, an extruder instrument (internal heat blender HAAK machine, Brabender, Germany) is used to melt the polymer and mix SiO_2_. The chamber is filled gradually with powder. The blended powder of SiO_2_/PEEK is gradually filled into the chamber until it melts and blends completely, which further gets converted into a brown-coloured homogenous blend at 320°C with 125 rpm. At this point, the rpm is decreased to 70 rpm and run for 5 minutes for uniform blending of the mixture.

Then, the dough of the PEEK material is collected using sharp knives and placed in a metal container. An electronic balance (of type A&D GF-600, Japan) is used to measure the weight of the collected PEEK material dough before loading it into the moulding chamber.

### 2.2. Heat Compression

A heat press (of type XLB-plate vulcanising machine, Germany) was used for the heat compression process. The heat component thermostat was set at 380°C. When the heat reached 200°C, the moulded polymer was introduced into the heat press chamber so that the heat was absorbed gradually. When the target temperature of 380°C was achieved, the pressure that started with 5 MPa increased gradually every 10 minutes until it reached 30 MPa finally. The mould was kept in the hot press chamber for 5 minutes. Afterward, the mould was taken out from the press chamber and kept in a cooling equipment (type Toyoseiki, Japan) at 30 MPa pressure and kept there until its temperature dropped to 200°C. Then, the pressure was released and the mould was put on a bench at 25°C°±°2 room temperature to achieve gradual cooling. After cooling, reconstruction of the mould and extraction of the sample from the frame was carried out and prepared for pretest calibrations.

### 2.3. Mechanical Tests

#### 2.3.1. Flexural Strength Test

The samples were constructed from a sheet with a length of 65 mm, a width of 10 mm, and a thickness of 3 mm). The test was carried out as per ASTM D790-03 by using a universal testing machine Instron with a velocity of (2 mm/min) at room temperature. The strength was computed as per the following formula.

- *S* = 3PL/2bd2
- *S* = stress MPa
- *P* = load at a given point on the load-deflection curve, N
- *L* = support span, mm
- *b* = width of beam tested, mm
- *d* = depth of beam tested, mm

#### 2.3.2. Tensile Strength Test

The samples were formed and cut as per ASTM D 638-Type3 (Standard 2011). The tensile test was carried out using a universal testing machine Instron 5567 tester at room temperature, with a gauge length of 25 mm and a cross-head speed of 5 mm/min.

### 2.4. Physical Tests

#### 2.4.1. Scanning Electron Microscope

An accelerating voltage of 10–20 kV of SEM type (LEO, model 1455VP, UK) is employed for microstructural analysis, while the energy dispersive X-ray spectroscopic analysis (EDX) is employed for determining the elemental composition of the materials. The main principle applied in spectroscopy is that each element possesses a unique atomic structure that can present a unique set of peaks with regards to its electromagnetic emission spectrum. EDX analysis relies on the interaction between a specimen for the elemental analysis and some source of X-ray excitation [16].

#### 2.4.2. Fourier Transform Infrared (Attenuated Total Reflection) Analysis (FTIR/ATR)

FTIR is employed for determining polymeric materials and it gives information based on the entire sample's physical state and chemical composition [17].

#### 2.4.3. Atomic Force Microscopy Examination

The AFM depends on the scanning technique and offers a high-resolution 3D image from the sample's surface. It is usually employed for identifying the surface morphology, topography, roughness, and distribution of particle size. A sharp tip that is present towards the end of the cantilever is employed in contact with the surface as well as the sample that has been displaced with piezoelectric scanners. The force present on the tip results in generating deflection that can be measured with the tunnelling capacitive or optical detectors. Zero standard pressure is used for the joint (to avert the chances of surface deformation).

#### 2.4.4. Contact Angle Measurement (Wettability Test)

A goniometer along with a charge-coupled-device camera and an image capture program employing Lab VIEW software are utilised to record contact angles.

### 2.5. Statistical Analysis

SPSS version 24 software was utilised in the statistical analysis of the data. Means and standard deviations were obtained for each test and unpaired *t*-test was used for comparison.

## 3. Results and Discussion

In this study, fillers are added to improve the PEEK characteristics. SiO_2_ Nanoparticles tend to have optimum characteristics that can be used in 3% with PEEK through the hot press method. This study aims to characterise PEEK as well as SiO_2_/PEEK composites, which can be prepared through melt blending procedures and hot press.

### 3.1. Transvers Strength and Tensile Strength

The SiO_2_/PEEK composite showed changes in the tensile strength and transverse strength versus PEEK alone. As per Table 1, a statistically highly significant increase with regards to the transverse strength was seen with the PEEK/SiO_2_ composite versus PEEK alone. With regards to the tensile strength, a statistically significant decrease in mean value was observed.

**Figure 2:**
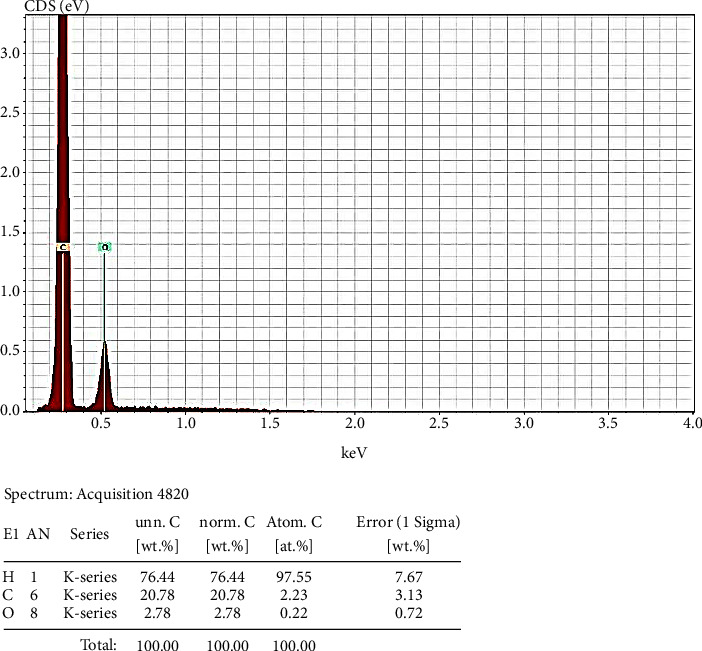
EDX image for the PEEK.

**Figure 3:**
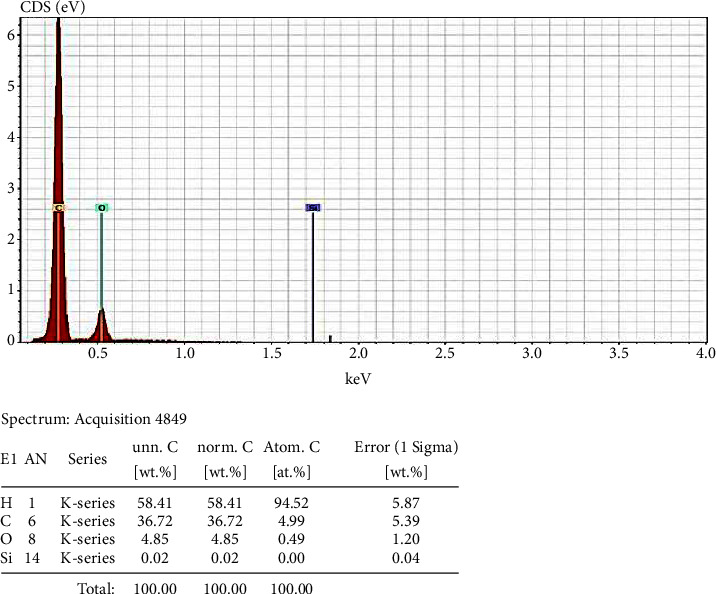
EDX image for the PEEK and SiO_2._

Figures 2 and 3 show the EDX spectroscopic analyses pertaining to PEEK and PEEK/SiO_2_ composite, respectively. These figures clearly show the elemental composition pertaining to the PEEK and SiO_2_/PEEK composite.

**Figure 4:**
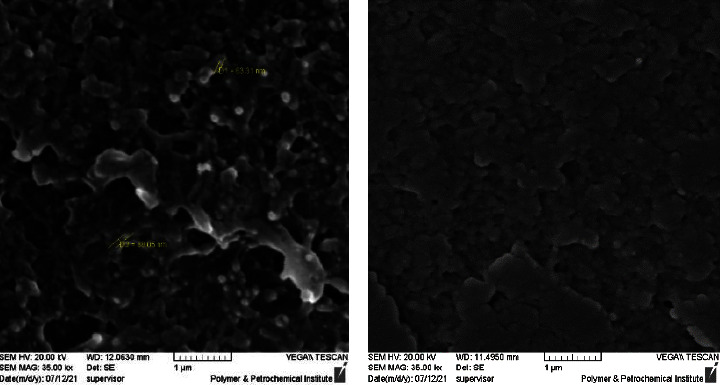
SEM image for PEEK/SiO_2_ and PEEK. (a) PEEK/SiO_2_. (b) PEEK.

**Figure 5:**
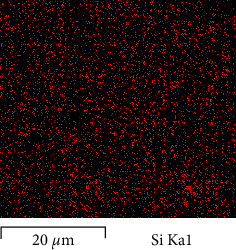
SEM/EDX image for SiO_2_ within PEEK.

As presented in Figure 4, uniform dispersion of SiO_2_ was noted in nanometres within the matrix based on the SEM image at MAG 35.00 kx pertaining to PEEK/composite and PEEK. As represented in Figure 5, this characteristic in the EDX map image also showed fair distribution pertaining to the Nano SiO_2_ particle within the PEEK matrix which is related to thorough and even mixing to the Nano by sonication then mechanical mixing for both Nano and powder then end with melt blending procedure.

**Figure 6:**
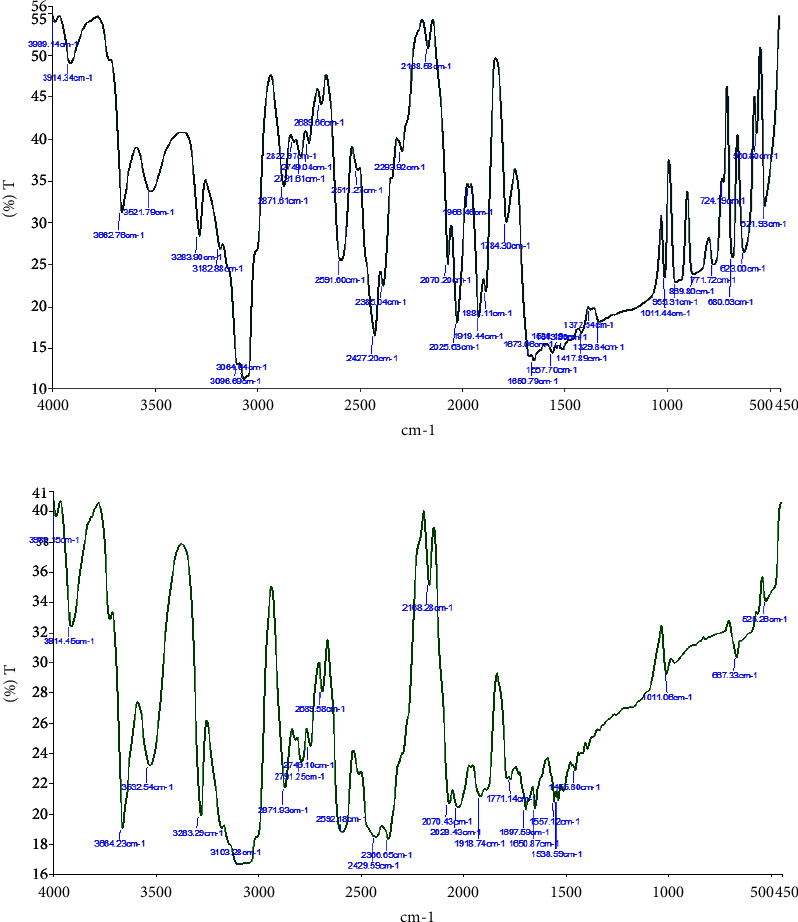
(a) FTIR transmittance spectrum SiO_2_/PEEK and (b) FTIR transmittance spectrum of PEEK.

As per the FTIR analysis result in Figure 6, the presence of SiO_2_ Nanoparticles is confirmed by the FTIR transmittance spectrum (400 to 4000 cm^−1^). To determine the presence of the O-H group, a broad peak in the range of 3000−3700 cm^−1^ was assigned. Similarly, at 1649 cm^−1^, a peak was seen that corresponded to vibration bending, which signifies the presence of the O-H stretching bond. Furthermore, the strong bands seen at 1093, 459, and 798 cm^−1^ were related to Si-O-Si stretching vibration bonding. Si‐OH (960 cm^−1^ and 1630 cm^−1^) or Si-O-Si (1094 cm^−1^, 798 cm^−1^, and 470 cm^−1^) were also detected.

**Figure 7:**
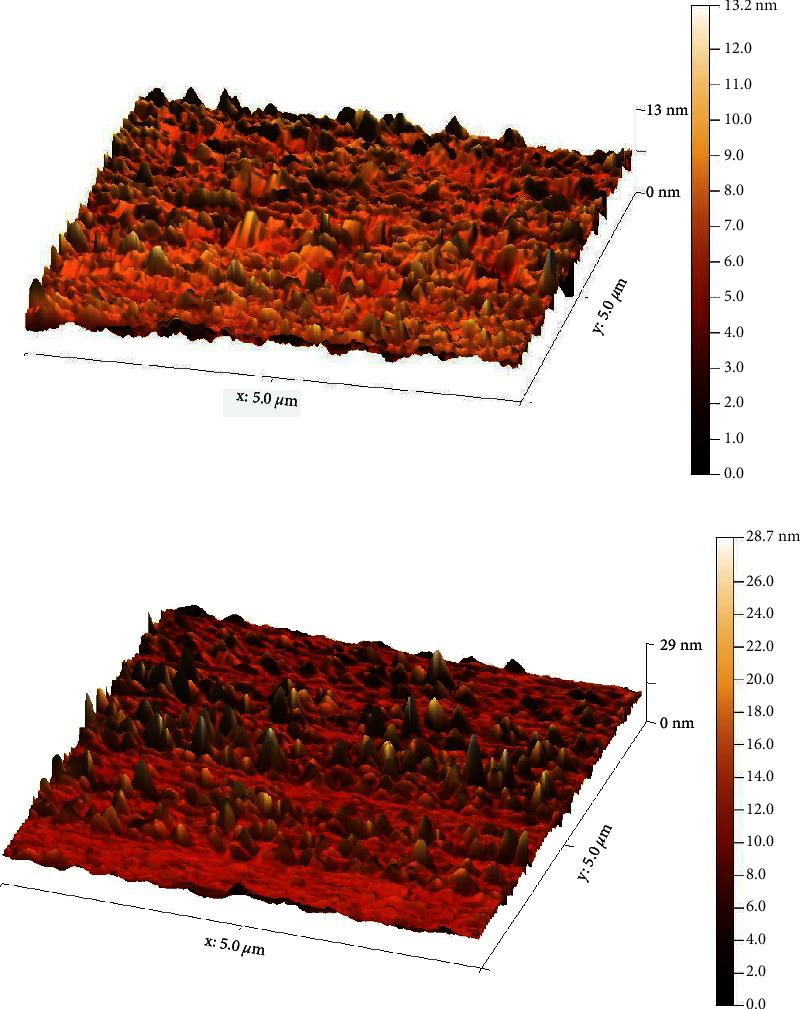
AFM picture of (a) PEEK/SiO_2_ and (b) PEEK.

As per the results of the AFM analysis seen in Figure 7, the surface characteristics of the PEEK/SiO_2_ composite and PEEK only showed marked differences. Between the two studied groups, there was a difference in the distribution and size of the spike.

Table 2 lists out Mean and StD for the surface roughness analysis as well as contact angle measurements pertaining to these tests. A highly significant decrease in the surface roughness and a statistically significant increase in the contact angle measurement were seen for SiO_2_/PEEK composite versus PEEK alone.

This study aims to enhance PEEK's properties with the addition of fillers. An optimum property was seen for PEEK with the addition of 3% SiO_2_ in Nanoparticles via the hot press method. The study aims to characterise PEEK as well as SiO_2_/PEEK composite prepared via melt blending and hot press methods.

The melt blending method is generally opted for synthesising Nanocomposites of a polymeric matrix. Usually, the polymer is melted first that let it to blend well with the required amount of fillers.

In this study, testing of the flexural strength pertaining to PEEK and SiO_2_/PEEK composite was carried out by complying with ISO178:2010. [18] As per the results, 3% Nano SiO_2_ was seen to clearly impact the transverse strength pertaining to PEEK. The addition of 3% SiO_2_ to PEEK is regarded to be optimum for fortifying PEEK and giving a clear enhancement in transverse strength versus PEEK alone which comes in line with Rikitoku et al. [13].

This was possible due to the large surface area pertaining to Nano size SiO_2_ which facilitates the creation of a large amount of interphase to offer a broad interfacial interaction as well as enhance the mechanical strength. The SEM images showed this and indicated the presence of SiO_2_ particles that are evenly distributed while maintaining minimal particle agglomerations.

With regards to the tensile strength, the addition of Nano SiO_2_ resulted in a decrease in tensile strength mean value, which is in line with the study of Wang et al. (2011), wherein the tensile strength relied on factors, such as the added concentration of Nanofillers to PEEK, the particles' dimension, and the way in which stress is transferred from the filler to the matrix [19].

This aspect can be related to the specific Nanomorphology particles that, beyond a concentration percentage in this study seems to be near to 4%, led to a dramatic toughness reduction and mechanical performance decline [20].

With regards to the Contact Angle Measurement, a variation was seen in the contact angle measurements pertaining to PEEK and Nano SiO_2_/PEEK Nanocomposites. With regards to SiO_2_/PEEK Nanocomposites added with 3% of hydrophobic Nano SiO_2_, there was an increase in the contact angle value versus PEEK alone. The surface roughness of the pure PEEK matrix could decrease when Nano SiO_2_ particles are added. This could be because of Nano SiO_2_ that are present on the surface of the Nanocomposites and are evenly dispersed within the PEEK matrix, as well as their small Nanoparticle size from 20–30 nm [21].

The small size of filler, particularly Nanosize, allows adhering to the resin matrix, thus offering a smoother surface finish, this result comes in agreement with Senawongse and Pongprueksa [22].

The limitation of this study was the limited tests used so more tests are required like compression and fatigue tests and the addition of another bioactive Nano to the PEEK/SiO_2_ composite could be studied.

## 4. Conclusions

By keeping the study limitation in mind, it can be said that 3% Nano SiO_2_ can be employed for synthesising SiO_2_/PEEK composite via the melt blend technique. Nano SiO_2_ can enhance the SiO_2_/PEEK composite's transverse strength while decreasing the hydrophobic characteristics pertaining to the surfaces with fair dispersion of nanoparticles within the matrix of PEEK.

## Figures and Tables

**Table 1:** Mean values and standard deviation of transverse strength MPa tensile strength MPa with Independent *T*-Test.

Test	Group	Mean (MPa)	std.	*T*	d*f*	Sig. (2-tailed)
Transverse strength	PEEK	2694.61	191.84	−10.06	18	*p* ≤ 0.001
	PEEK and 3%SiO_2_	3503.02	166.64			

Tensile strength	PEEK	97.62	3.25	23.415	18	*p* ≤ 0.001
	PEEK and 3%SiO_2_	63.69	3.22			

**Table 2:** Mean values and standard deviation for surface roughness and contact angle data of pure PEEK and SiO_2_/PEEK nanocomposites.

Groups	Surface roughness (nm)		Contact angle	
	Mean	std.	Mean	std.
PEEK	1.23	0.01	71.01	4.57
SiO_2_/PEEK	0.66	0.01	81.78	0.84
Sig (2-tailed)	*p* ≤ 0.001		*p* ≤ 0.001	

## Data Availability

The data that support the findings of this study are available from the corresponding author upon request.
